# British Medical Association

**Published:** 1874-10-01

**Authors:** 


### BRITISH MEDICAL ASSOCIATION.

*From the Philadelphia Medical and Surgical Reporter.*

Norwich, Tuesday Evening, August 11.

The British Medical Association commenced its forty-second annual meeting to-day. The proceedings commenced with a special service in the Cathedral, the preacher being the Rev. Canon Heaviside. In the afternoon a council meeting was held, and this evening the inaugural general meeting was held, when the retiring President, Sir William Fergusson, resigned the chair to the President elect, Dr. Edward Copeman.

The President elect, in his inaugural address, recalled the fact that the celebrated Sir Thomas Browne was a Norwich physician; that the eccentric Dr. Messenger Monsey was the son of a Norfolk clergyman, and well known as Sir Robert Walpole's Norfolk doctor; that one of the Aldersons practiced in Norfolk as a physician; that Dr. John Kaye, or Caius, of Cambridge notoriety, was born at Norwich, in 1510; and that the late Mr. Martineau was surgeon to the Norfolk and Norwich Hospital for many years, and was regarded as one of the greatest lithotomists of his day. He might add the names of Rigby, Dalrymple, Lubbock, and Crosse, the last having been one of the foremost of provincial surgeons, and one of the founders of the British Medical Association. Norwich had a noble provincial hospital, more than one hundred years old, containing a museum in which were displayed all the stones removed by operation since its foundation, as well as many unique specimens of morbid anatomy.

Wednesday, Aug. 12.

The British Medical Association continued its proceedings to-day, an address on "Medicine" being delivered by Dr. J. Russell Reynolds, Professor of Medicine in University College, and Physician to University College Hospital. Dr. Reynolds argued that if we would know the present condition of medicine and pathology, we must see whence it had come and whither it was tending. It was not his intention to attempt to furnish an account of the details of recent scientific works on medicine, but rather, by an examination of our past and present relations to four great propositions, or, if he might use the term, articles of creed, to show how they had affected our modes of investigation in the past, how they are governing or guiding our labors now, and what were the results which we saw coming from the now-existing and prevailing tendencies of thought. The four articles of creed to which he referred as influencing for good and for evil the progress of scientific pathology, were a belief, first, in life; secondly, in man; thirdly, in individuality; and, fourthly, in the specific character of disease. By losing sight of or underrating the great primary fact of life, we deprive ourselves of the information to be gained from a study of subjective symptoms, we often misdirected our therapeutic efforts, by eliciting vital action rather than conserving vital force, and we lost sight of many of the most important causes of disease. By failing to see the specialty of the nature of man, we underrated or ignored much of the etiology of human suffering, we were often misled by the results of observations upon animals, and we were in danger of misinterpreting the facts of the most serious maladies which might afflict our fellow-creatures. By disregarding the individuality of man, we were in danger of again, and in another way, losing a due appreciation of the causation of disease, and of overrating the value of statistics, and of being led away by their apparent precision, which existed only with regard to masses. By an unsound application of the idea of the specificity of disease, we might, on the one hand, sweep away distinctions which were facts of pathology, and, on the other hand, lay down lines of demarcation which were unreal. It was, he thought, obvious that the science of medicine was in a state of change, as well as of progress. It had not yet arrived at the degree of exactness which might be found in the simpler sciences; but those who were devoting themselves to its pursuit were pressing toward that end, and each was endeavoring so to throw his mite into the treasury of knowledge that a scientific ordination of the facts of pathology might hereafter be a possibility. But the facts were so diverse in kind, so varied in appearance, and so bewildering in their complexity of form, that we must wait, and, perhaps, wait long for their due arrangement. A true science of pathology could not be until the knowledge of all individual workers was the common property of all. In the meantime, we must learn to labor and to wait; and we must, least of all, commit ourselves to any one particular school or line of thought, but rather cultivate that habit of mind which had "a look southward, and was open to the whole noon of nature." In our recognition of the specialty of disease we had carefully to examine the nature and the sources whence they came, of all the contaminations by which the stream of life might be damaged or defiled. They might be puzzling, hard to separate, and still harder to trace to their source; they might be complicated in their effects, and so mixed together that again and again we might be baffled in our attempts at their analysis; but what we wanted was work, not on any preconceived theory, but work which should faithfully follow onward and trace backward the line of sequence of events, and eventually yield, as it had done before, a real knowledge of the facts we wished to know. Again, with regard to the individuality of man, we must strive to show whence each life had come, the nature of its source, the conditions of its youth, the struggles of its onward progress, the valleys through which it had passed, and the rocks it had to "overleap or rend." We might be often perplexed in our attempt to solve the problem which it presented, but we must accept the facts of their existence and of their variety. We must know all that made up individual life if we would understand its ills. We must go back to its source, and look around at all through which it had had to pass or work its way, if we would know how to understand or arrest the troubles it might meet. Again, with regard to man, it was not until we recognized in him his true relation to the world which lay around him that we should duly understand his sorrows, or whence they came, in such a way as to prevent them or relieve them when they were thickly hedging him about. Lastly, we must duly appreciate the great fact of life, the mystery of mysteries, which underlay all our knowing, and overarched us sometimes with light, sometimes with impenetrable gloom. When we tried to realize it, we seemed to be on the surface of a boundless ocean; above us the sky, now dark with cloud and rent by storm, now lit with sunbeams or trembling with the pale light of stars, beneath us a mighty deep, with its unknown treasures, mysterious currents, and "sense of unfathomable danger." Sometimes we saw the land, and pressed toward it, but again the night came down, the horizon line was lost. We were poised between two worlds, and although we might strain our eyes to see and our ears to hear, we might find no token to tell us of the points in which they met, no glimmer of the unseen things which severed them. But let us thank God that we were not left alone, and that in such darkness there often arose light.

The address was frequently applauded, and on the motion of Professor Hughes Bennett, a unanimous vote of thanks was tendered to Dr. Reynolds. Professor Bennett observed that the occupation of the medical man and physiologist was to determine the laws which governed vital properties. This task presented enormous difficulties, and how were they to be overcome? He contended that medical men were justified in making use of the inferior animals, with a view to the solution of the problem. He considered that many existing difficulties and mysteries might be overcome with work, if workers were found, and if those workers were properly remunerated. Dr. Bateman seconded the vote of thanks. It was agreed that the summer meeting of the Association should be held in Edinburgh, and Sir R. Christison was elected President for 1875.

In the Public Medicine Section, over which Mr. W. H. Michael presided, Dr. Rogers, as President of the Poor Law Medical Officers' Association, made a statement to show that where medical relief was stinted the general expenditure on Poor Law relief was heavy.

Dr. Beverley read a lengthened paper on Hospital Hygiene, illustrated by reference to the Norfolk and Norwich Hospital.

A vote of thanks was accorded to Sir W. Fergusson, the retiring President, for his services during the past year, and he was unanimously elected Vice-President for life.

A report was presented from the Council, which stated that the Association now numbered between 5,000 and 6,000 members, and its financial condition was also satisfactory. The income from all sources last year was £8,511, and the debt of the Association was reduced to £212, with a prospect of total extinction this year. Under these circumstances the Council proposed that £200 should be granted in aid of researches in medicine and allied sciences. The Council stated that an invitation had been received that the next country meeting of the Association should be held in Edinburgh. After some discussion, the report was adopted, and Mr. F. Fowkes was re-elected Secretary for 1874-5. The meeting was then adjourned to Thursday.

In the evening there was a *soiree* in St. Andrew's Hall.

THURSDAY, Aug. 13.

This morning the British Medical Association resumed its sittings, when an address on Surgery was given by Mr. W. Cadge, F. R. C. S. Surgeon to the Norfolk and Norwich Hospital. Mr. Cadge observed that the novelties of surgical practice introduced during the last year or two were scarcely important enough to constitute a theme. They were chiefly comprised in Esmark's bloodless system of operating and Dittel's elastic ligature. Concerning these, he would only say that the first was not a novelty, having been practiced by one of the members of the Association many years since; the second was, in his opinion, more curious than useful, and not worthy a place either in the records or the practice of surgery. There was one great subject which, if not exactly a novelty, was of recent time, and was still waiting for a solution, both as to its facts and as to the theories held to account for its facts—he meant the germ theory of putrefaction and antiseptic surgery. This subject he considered of surpassing importance and interest. Underlying, if not undermining much of the existing fabric of surgical pathology and practice, it could not be too often brought to the bar of professional criticism, for on its right solution, whether it was true or not, depended a multitude of points in daily practice, and even, probably, the lives of many of our fellow-creatures. The remainder of

Mr. Cadge's paper was devoted to an elaborate examination of stone diseases, which occasions an annual mortality at the rate of one in 42,744 of the population in Norfolk, as compared with one in 425,525 in Cheshire. Incidentally alluding to the importance of milk for the support of young children, Mr. Cadge observed that it would be a glorious result of statecraft if—instead of the futile wrangling over the sale of fermented liquors, which had wrecked one powerful government, and, by the disappointment of greedy expectants, had gone far to sap the popularity of its successor—by some equitable enactment, those who possessed and occupied the land should be held responsible for the production in sufficient abundance for the wants of the poor of that which was now a costly luxury, but which nature pointed out to be the chief—he might say the only—need of early childhood. The local influences which contributed to renal and vesical calculus in Norfolk were, Mr. Cadge thought, the universal consumption of malt liquor, the constant daily use of exceedingly hard drinking water, and the accumulated effect of hereditary predisposition.

A cordial vote of thanks was accorded to Mr. Cadge for his address.

Professor Hughes Bennett made a statement as to the antagonism of medicines—hydrate of chloral and strychnia, sulphate of atropia and calabar bean, hydrate of chloral and calabar bean, sulphate of atropia and meconate of morphia, meconate of morphia and infusion of tea, meconate of morphia and theine, meconate of morphia and caffein, meconate of morphia and quaraine, meconate of morphia and infusion of coffee, extract of calabar bean and strychnia, hydrate of bromal and atropia, etc. The statement was received with much attention and elicited a hearty vote of thanks.

Approval was given to measures taken for the incorporation of the Association under the Companies Acts, 1862 and 1867.

Sir James Paget delivered an able address yesterday afternoon, as President of the Surgery Section. He observed that in the present day we overvalued the blood, and estimated too cautiously the loss of it. There were few persons in the room who might not be bled to fainting and to-morrow be almost unconscious of it; perhaps in this week of hospitalities they might even be the better for it. [A laugh.] Referring to the use of mercury, Sir James observed that in his youth mercury was largely administered. It probably did good in a large number of cases of which the real nature was not at the time discerned, and in a large proportion of the chronic diseases of internal organs which we now assigned to syphilis. Years ago there was no suspicion that syphilis affected any but external parts. We knew now a multitude of syphilitic affections of the liver, of the lungs, of the spleen, and many more still of the nervous system, which formerly were vaguely put down to chronic inflammation of unknown origin, or to tumors, thickenings, and productions of substances which needed to be absorbed. At the present time we were rather apt to think that pathology should be the guide of therapeutics, while there was a large number of cases in which therapeutics should rather be the guide of pathology. The fact that a medicine cured a given disease was as much a fact, and quite as significant a one as the employment of a chemical test for discerning the nature of a solution. It could be repeated from time to time, and with the same results. There was hardly anything in the chemistry of complex bodies more sure than that quinine cured ague and a large number of periodic diseases. As with quinine, so with mercury. If in his youth the value of therapeutic tests for indicating disease had been fairly estimated, we should have come many years sooner than we did to a knowledge of the syphilitic nature of a large number of internal chronic diseases. We were, he believed, too much under the guidance of what might be justly called inferential therapeutics. Because we knew something of pathology, we might, therefore, proceed at once from pathology to the knowledge of the remedies of disease. It was a fair method of study if it were not carried to excess, but it should be studied side by side with the other fact, that therapeutics might just as fairly be a guide to pathologic knowledge.

In the Public Medicine Section, today, Mr. W. H. Michael again presided. A discussion arose on a paper read by Dr. Beverley on the preceding day on hospital hygiene, with especial reference to the Norfolk and Norwich Hospital, in which a large amount of pyæmia has prevailed lately. Dr. Beverley recommended the construction of a series of single-storied buildings for the reception of such patients, care being taken to secure thorough ventilation.

Mr. Sympson, of Lincoln, made a statement as to defects in the Lincoln County Hospital, where pyæmia and erysipelas had also prevailed to some extent, the ventilation being imperfect. North winds were found to be injurious, but it was not the same with east winds, as Dr. Rumsey had feared to be the case in a London hospital.

Dr. Druitt expressed his opinion that when once a hospital became infected, the best course was to pull it down.

Mr. Barwell observed that the economic aspect of the question must be considered. In the case of the Norfolk and Norwich Hospital he attributed present difficulties to injudicious additions to the original building. There ought to be no place in a hospital where air could stagnate, and the staircase ought not to be in the body of the building.

Dr. Falconer observed that it was a mistake to speak of air as if it were under our perfect control. Air could not be driven about as we liked. At the same time every facility should be given for the ingress and egress of air from a dwelling. An attack of erysipelas had broken out in St. George's Hospital. Washing was stopped in the hospital, and the disease disappeared. He thought it was possible to have movable roofs to hospitals; these roofs might be periodically raised, and a thorough ventilation secured. By such a system as this it would not be necessary to pull down old hospitals, although they had a tendency to become contaminated.

Mr. C. B. Fox said the walls of old hospitals had a tendency to become saturated with organic matter. Had any measures been adopted to remove this organic matter at Lincoln or Norwich?

Dr. Copeman said he remembered a very serious outbreak of erysipelas in the Norfolk and Norwich Hospital, before recent additions to the hospital were made. So severe was this outbreak, that operations were suspended for a time. Afterwards there was a remarkable immunity from erysipelas in the hospital. One roof in the hospital had been found to contain a large quantity of impure air.

Mr. Hutchinson remarked that at Leeds a splendid new hospital had produced a large amount of pyæmia. Care in dressing wounds and isolation of contagious cases had been found to be productive of the best results. Erysipelas often originated *de novo.* He believed also that pyæmia originated from local contagion rather than from the air.

Dr. Fussell deprecated pupils or surgeons passing from fever wards to accident wards, or from post-mortem examinations to accident wards.

Dr. Beverley replied. He stated that measures were being adopted to cleanse one of the walls of the Norfolk and Norwich Hospital. The question of economy must of course be considered, but still human life must not be sacrificed, and it would be better to have a small and thoroughly efficient hospital, than a large and inefficient one. Some other points having been noticed by Dr. Beverley, the discussion closed.

Dr. C. J. Fox read a paper "On Water Analysis; as it should and should not be performed by the Medical Officers of Health." Dr. Fox observed that the elementary principles upon which the greater part of the work of the Medical Officer of Health was based might be said to be the prevention of water pollution and of air pollution with the products of decomposing filth. The examination of drinking water formed a very important portion of his duty in his crusade against preventable disease. The most rough and ready way which has been employed for ascertaining whether or not water was polluted with organic matter was to partly fill a clear bottle with a sample of it, and having violently shaken the same, to take a hearty sniff at the air of the bottle which had been agitated in the water. If the air smelt sweet and fresh, the absence of an injurious amount of organic matter was inferred, and *vice versa*. It should be borne in mind that the existence of an unpleasant odor or taste about the water from a well sunk through certain kinds of clay was no proof of the pollution of water with organic matter. Water, if allowed to remain long in contact with certain kinds of clay, acquired such an objectionable smell as to be at times quite undrinkable, and yet might not at the same time contain an amount of organic matter which would warrant its condemnation. A well of this kind could be made to furnish excellent water by the frequent withdrawal of its contents, or, if that was not practicable, by the filling up of the dug portion of the well, and by drawing the supply solely from the bore pipe. Dr. Fox, in closing a paper of considerable length, said his object in bringing the subject of water analysis before the Association, which numbered among its members so many Medical Officers of Health, was the hope that some uniform plan of examination might be adopted by all.

Mr. A. Haviland made some observations on the geographical distribution of disease within the area of the counties of Northampton, Leicester, Rutland, and Bucks. He expressed an opinion that typhoid fever was contagious, although he knew that it was a moot point. Consumption prevailed to a great extent in the valley of the Nene, especially among women, who lived less out of doors than men. Every Northampton shoemaker was fond of poaching and country life, but the poor women remained at home. He complained that one medical man had returned scarlet fever as sore throat, and that the fever had spread in consequence. He thought strict accuracy was essential in all such matters.

After a short discussion, the section adjourned.

The usual public dinner was held in the evening, in St. Andrew's Hall. The chair was occupied by Dr. Copeman. There were about 300 guests, and the hall presented a brilliant appearance. The Duke of Connaught could not be present, in consequence of other engagements. It was suggested that next year there should be a section for military and naval surgery.

FRIDAY, August 14.

This morning the British Medical Association continued its sittings. An address in obstetric medicine was delivered by Dr. J. Matthews Duncan, physician to the Royal Maternity Hospital, and Lecturer on Midwifery in the School of Medicine, Edinburgh.

In the Public Medicine Section, Dr. Drysdale, senior physician to the Metropolitan Free Hospital, and physician to the North London Consumption Hospital, submitted a paper on the influence of tobacco on public health. Dr. Drysdale observed that in the midst of all cheering signs of the march of scientific civilization, it was curious to remark that the custom of tobacco-smoking became every year more prevalent. He did not think that alcoholic intoxication was so prevalent as it used to be, although it was still terribly prevalent; but he expected no one to doubt his assertion that tobacco was becoming much more extensively consumed, and that even by the highest classes. Quite recently the leading medical journal, edited as it was by writers of great literary merit as well as great professional eminence, recommended the use of tobacco as a necessary for our troops on foreign service, thus classing it with the hygienic remedies useful in malarious and tropical districts. He therefore almost feared that any one accusing tobacco of doing any harm to the public health might not impossibly run the risk of being charged with the madness of the great Spanish hero of Cervantes, and be reputed as running a tilt against windmills. He had seen, however, in the course of his life, so many cases of disease, which he, rightly or wrongly, had attributed to the use of tobacco, that he felt it his duty to say a word on the negative side of the question. It was not above a month or two since that he saw, in the course of one week, two cases of complete blindness, accompanied by atrophy of the optic disc, in men, entirely due, he was sure, to the use of tobacco. One of these patients was of the age of twenty-seven, and had been a most extensive smoker for some six years, consuming, he said, an ounce of Virginia tobacco daily. The other unfortunate young man was only twenty-four years old, and he had been in the habit of chewing constantly as well as smoking. His amaurosis was quite similar in character with that of the other patient. Affections of the gums and tongue were very frequently seen in old smokers. The tongue looked as if it had been painted with a solution of nitrate of silver in some cases; in others there was lividity of the gums and great duskiness of the fauces. Among the poorer classes tobacco-smoking and chewing often made a man's mouth a deplorable spectacle. Dyspepsia and diarrhœa were more frequently caused by smoking than many believed, and Sir W. Jenner used to say, and he (Dr. Drysdale) thought with truth, that the use of tobacco disposed to palpitation of the heart, prolapse of the rectum, etc. Whatever might be thought of this view, he could cordially subscribe to the opinion which ascribed to tobacco many of the cases of *malaise* and cachexia of men who would otherwise be in excellent health. Tobacco-smoking had left the tap-room, and now even the Throne itself was not destitute of the smell of tobacco. Were the modern Turks or Germans to go on smoking from morning till night, as they did, and consuming Virginia tobacco or Cavendish as we did in England, they would indeed present a deplorable spectacle of public want of stamina. Fortunately, however, German and Turkish tobacco was almost devoid of noxious properties. In Russia, Turkey, and some other nations, women had already begun to invade the smoker's domain, which in England they had at present left unexplored, to the great satisfaction of all who valued good complexions in their countrywomen. In Russia elderly ladies asked smokers for a light for their cigarettes or cigars, and taught their daughters to blacken their teeth and injure the purity of their breath by inhaling the fumes of the burning weed. He could not help thinking, with the physicians of more primitive times, that simple diet, with absence from stimulants and narcotics, would, to the end of all time, be found the greatest friends to virtue, beauty, and good health. Railway traveling, always so uninviting, was now rendered much more so by the stale smell of tobacco smoke. The whole of Europe, it had been remarked by Professor Mantejezza, was fast being turned into a cigar divan; and it became every day more difficult for a male of average strength of mind to assert his liberty to refrain from tobacco if he went into society at all. He (Dr. Drysdale) thought, then, that it was time for such practitioners of the healing art as had anything to say about tobacco, to speak out. For his part he charged tobacco with causing blindness, palpitation of the heart, paralysis, diarrhœa, and diseases of the teeth and mucous membrane of the mouth and tongue. He alleged that it was a foe to cleanliness and good manners. He knew that it was injurious to workers in tobacco factories, and he therefore contended that it was not a true luxury, and never a necessary. Tobacco might be used for the treatment of asthma, but to admit tobacco-smoking, chewing, and snuffing, into the list of the luxuries of a refined and wealthy age, was, in his opinion, a violation of the laws of public hygiene.

The President (Dr. Bateman, of Norwich,) said he thought the question of sewage was not merely one of local interest, but of imperial interest. In Norwich the citizens had been experimenting at heavy cost in the matter of sewage and irrigation, and they would still have to expend a further sum, and at the same time take a leap in the dark. In Norwich the ratepayers were, in fact, in the position of pioneers who were spending a large amount of money for the benefit of others.

The section then adjourned.

The concluding general meeting was held this afternoon ; Dr. Copeman presiding. A discussion arose upon a report presented by a committee upon qualification and state medicine. Dr. Rumsey proposed that the question should be referred to Dr. Lyon Playfair, M. P., Mr. G. W. Hastings, and the Rev. Professor Haughton, of Dublin. The report was rceived, with power to the committee to adopt the suggestion of Dr. Rumsey. A grant in-aid committee was appointed to carry out a recommendation that £200 should be voted for scientific researches. Thanks were voted to the East Anglian Branch of the Association and the local members of the medical profession, for their reception of the Association. Sundry other votes of thanks were accorded to the Mayor and Corporation of Norwich, to Mr. J. J. Colman, M. P., to Lady Lothian, Lady Crossley, and other ladies and gentlemen, for courtesies extended to the association. A cordial vote of thanks was also given to the president, Dr. Copeman.

The proceedings then terminated, although sundry excursions have been arranged to places and scenes of local interest.

